# Infectious Tolerance as Seen With 2020 Vision: The Role of IL-35 and Extracellular Vesicles

**DOI:** 10.3389/fimmu.2020.01867

**Published:** 2020-08-26

**Authors:** Jeremy A. Sullivan, David P. AlAdra, Brian M. Olson, Douglas G. McNeel, William J. Burlingham

**Affiliations:** ^1^Department of Surgery-Transplant Division, School of Medicine and Public Health, University of Wisconsin, Madison, WI, United States; ^2^Departments of Hematology and Medical Oncology, Urology, and Surgery, Emory University School of Medicine, Atlanta, GA, United States; ^3^Department of Medicine, School of Medicine and Public Health, University of Wisconsin, Madison, WI, United States

**Keywords:** IL-35, extracellular vesicle, infectious tolerance, exosomes, immunotherapy

## Abstract

Originally identified as lymphocyte regulation of fellow lymphocytes, our understanding of infectious tolerance has undergone significant evolutions in understanding since being proposed in the early 1970s by Gershon and Kondo and expanded upon by Herman Waldman two decades later. The evolution of our understanding of infectious tolerance has coincided with significant cellular and humoral discoveries. The early studies leading to the isolation and identification of Regulatory T cells (Tregs) and cytokines including TGFβ and IL-10 in the control of peripheral tolerance was a paradigm shift in our understanding of infectious tolerance. More recently, another potential, paradigm shift in our understanding of the “infectious” aspect of infectious tolerance was proposed, identifying extracellular vesicles (EVs) as a mechanism for propagating infectious tolerance. In this review, we will outline the history of infectious tolerance, focusing on a potential EV mechanism for infectious tolerance and a novel, EV-associated form for the cytokine IL-35, ideally suited to the task of propagating tolerance by “infecting” other lymphocytes.

## Introduction

The concept of “Infectious Tolerance,” originally proposed in the early 1970s by Gershon and Kondo (1) and expanded upon two decades later by Herman Waldman (2), pre-dated the discovery of specialized T regulatory cells (Tregs) and Foxp3 by several years (3–5). The early work elegantly described the cooperative activity of T cells with their antibody producing cousins, the B cells in developing tolerance. Using various murine models, they were able to develop and break tolerance by manipulating the contributions of the T cell in developing and producing antibody from the B cell (1). The later work focused on the ability of T cells, once tolerized, to transfer or “infect” transplantation tolerance into a brand new set of T cells (2, 6, 7). While the work of Sakaguchi et al. (8) and Fontenot et al. (9), led to the description of a specialized CD4 T regulatory cells of the immune system, the earlier work by Hall et al. (10) and Illano et al. (11) drove home the idea that a subset of CD4^+^ T regulatory cells could be responsible for initiation of peripheral tolerance. What wasn't immediately clear, is what caused the “infectious” nature of tolerance enforced by Tregs. That is to say, the transferable nature of immuno-regulation that Qin et al. (2) had shown so clearly in 1993, whereby one set of (induced) regulatory T cells could convert naive T cells such that they would also become tolerant, lacked any mechanism. The problem was that the type of immuno-regulatory cytokines known at the start of the 21^st^ century, including TGFβ (12–15) and IL-10 (16, 17), were known as powerful primary immuno-suppressives, but neither were known for their ability to induce regulatory activity in other, non-Treg cell types. Therefore, TGFβ, and IL-10 couldn't adequately describe the “infectious” nature of infectious tolerance. The more recently described member of the IL-12 cytokine family, IL-35, is a highly immunosuppressive cytokine that has been implicated as an agent for infectious tolerance (18). IL-35, unlike IL-10, however, has recently been associated with a unique mechanism of action, one that involves its association with extracellular vesicles (EVs) (19). The EV nature of IL-35 secretion and function, lend it to be a favorable candidate cytokine to propagate an immuno-suppressive signal in a non-specific, infectious manner. In this manuscript, we will review the tremendous impact of EVs upon transplant immunology in recent years and consider the role of the cytokine IL-35, ideally suited to the task of propagating tolerance by “infecting” other lymphocytes.

## The Extracellular Vesicle Impact in Transplantation Biology

Two major publications from 2016 provided a potential conceptual change in transplantation biology. Parallel studies by Benichou (20) and Morelli (21) contradicted the long held belief (22) that dendritic cells (DC) within the allograft were themselves directly responsible for inducing the massive immune response to tissue/organ transplants—the phenomenon of acute rejection (AR). Instead, graft passenger DC turned out to be merely the source of the problem. The real culprit driving AR turned out to be extracellular vesicles (EV), or more specifically, exosomes (30–150 nM in diameter) produced by these very same DC. In the first hours after transplantation, these vesicles began to decorate the surface of so many recipient DC with donor MHC antigens, along with co-stimulation (for e.g., CD86 also found on the vesicles was co-expressed with allo-MHC on the host DC surface), that allo-reactive T cells throughout the body became alerted to the graft. Furthermore, this multiplier effect of exosomes did not require the passenger DC to leave the graft. DC of highly immunogenic skin graft, were “trapped” in the graft because of lack of vascular input until day 4. Nonetheless, they provided a prolific source of exosomes containing abundant alloantigen that escaped the graft via lymphatics within 24 h of skin placement, decorating host DC in local lymph nodes, and activating host T cells (20). Remarkably, and almost unnoticed in light of the demonstration that “semi-direct,” rather than the *in vitro* standard of “direct” pathway allo-activation, was the actual cause of AR, was that T-helper function on the so-called “indirect” pathway of allo-peptide recognition was also working smoothly. The uptake of exosomes by host DC did not interfere with provision of “help” in the form of TNFα and IL-2 from T helper cells recognizing allo-peptide/MHC complexes on the same host DC. Thus, the “semi-direct” allo-reactive T cells were sustained, such that AR was carried through to completion, with severe and irreversible damage to the graft. One question that arose from these observations was the extent of the relationship between the nature of the indirect response, in the form of altered co-stimulatory molecule expression, and the impact this would have on a potential semi-direct AR response.

To answer this question, we decided to investigate other ways that DC EVs could become involved in transplantation immunity. We had long been interested in the phenomenon of non-inherited maternal antigens, and their effect upon host allo-response (23–25). We found that not only could DC-derived EVs cause massive activation, but alternatively, by stimulating networks of immune regulation, they could cause a type of rapid but “un-helped” AR that would attenuate quickly. Should the host be able to get past this accelerated acute rejection response, as is the case in most kidney transplantation due to early immunosuppressive drug therapy, it would be followed by long-term graft protection. How this occurs is a tale of two very different components of maternal DC EVs.

**Figure 1:**
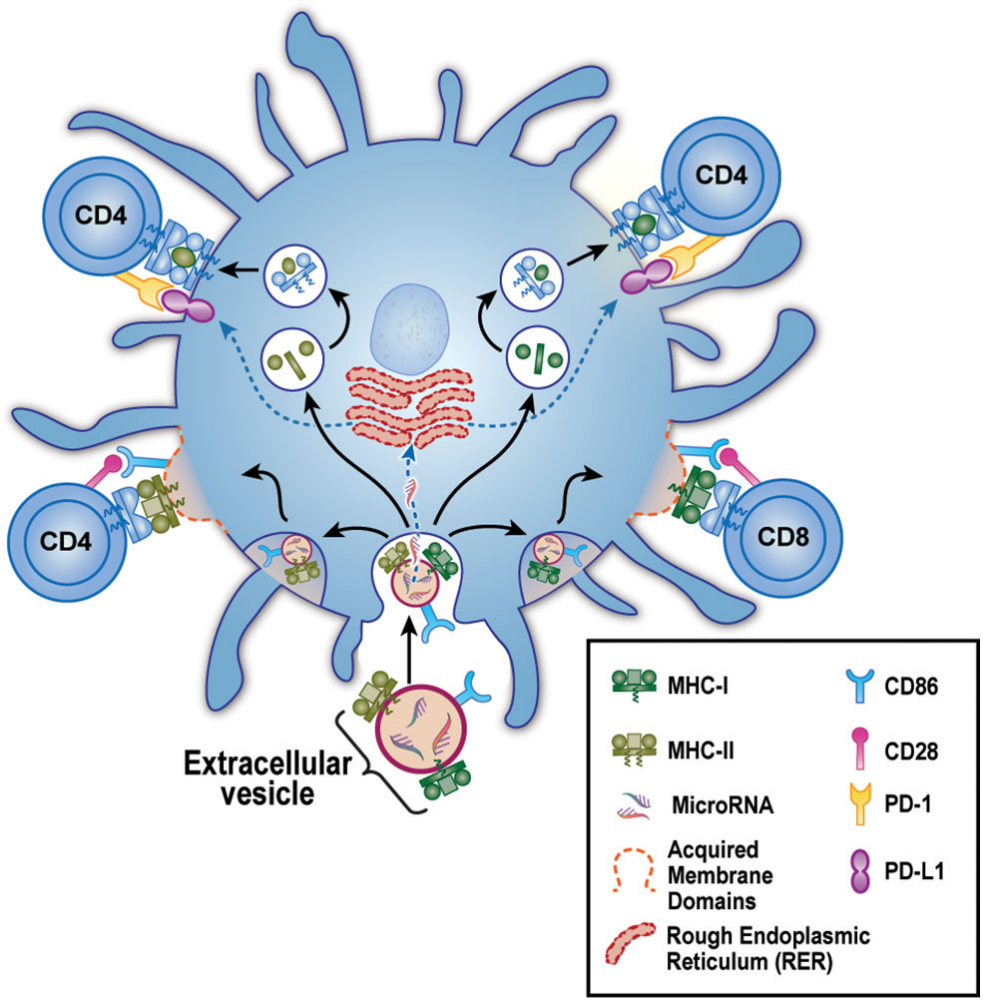
Model for EV-Associated Dendritic Cell “split” tolerance. An extracellular vesicle (EV) derived from maternal micro-chimerism [or from certain allograft types] is taken up by a host dendritic cell (blue). These EV contain microRNA and express surface membrane-bound allo-MHC class I (green) and II (olive), along with the co-stimulatory molecule CD86. After uptake, the microRNA escape to the endoplasmic reticulum where they guide production of PD-L1 (purple). The allo-class I and class II are either **(bottom)** preserved intact and re-distributed as components of acquired membrane domains (dashed red lines) containing CD86, or **(top)** broken down in lysosomal vesicles to peptides that are loaded onto “self” MHC class II. These 2 forms of allo-antigen presentation, semi-direct (lower left and right), and indirect (upper left and right) are kept separate by the host DC, allowing positive co-stimulation of semi-direct pathway, allo-specific host CD4 and CD8 T cells via CD86-CD28 (pink) interaction. Yet even as these productive interactions are occurring, the CD4 T helper cells are being strongly inhibited by PD1 (yellow) interaction with the negative co-stimulator PD-L1. The net result is limited acute rejection in the short term, followed by long-term protection of the allograft from chronic rejection (“split” tolerance).

In 2017, we reported in a mouse breeding model that EV delivery of maternal MHC and CD86 to the neonate's DC had a very peculiar effect on the immune response of the offspring (26). On the one hand, as predicted by the work of the Benichou and Morelli labs, these maternal (allo) exosomes could greatly increase the frequency and rapidity of acute rejection, due to intact maternal MHC and co-stimulation acquired from the exosomes and re-expressed by the neonate's own DC. This could explain the enhanced onset of acute rejection episodes after transplantation of a kidney from a live-related sibling that expressed the non-inherited maternal HLA antigens (NIMA), as reported previously (24). On the other hand, and contrary to a standard acute rejection of the kind studied by Benichou and Morelli, we found that in mice born with NIMA^+^ DC micro-chimerism, the same maternal exosomes that “cross-dressed” the offspring's DC with her MHC antigens could also induce high levels of PD-L1 expression on the same cell (Figure 1). This effect was likely mediated via microRNA content of the EV, as the EVs themselves appeared to lack PD-L1 protein (26). Furthermore, the PD-L1 produced by the EV “cross-dressed” host DC was not uniformly expressed, rather PDL-1 protein was excluded from areas on the cell surface expressing the acquired maternal antigen. Thus, two distinct areas on the mature offspring's DC surface were created: (1) patches where not only the maternal MHC class II, but also the co-stimulatory molecule CD86, were located, and (2) areas outside these patches where a more uniform distribution of PD-L1 was observed. This meant that the semi-direct- allo-recognition pathway for the NIMA was preserved, just as Morelli et al. (27) and Marino et al. (20) had envisioned for acute allograft rejection. However, unlike the case in “classical” acute rejection, T helper function from the indirect allo-recognition pathway would be lacking. That was because, while the response of a direct pathway T cell clone to allo-antigen expressed by exosome-acquiring pDC and mDC was enhanced as predicted by Morelli and Benichou, helper function provided by an indirect pathway T cell clone, reacting to a processed allo-peptide on the same DCs, but outside the “semi-direct” zone, was markedly inhibited in a PD-L1-dependent manner (Figure 1) (26). These results supported another clinical finding, namely the reduction in chronic rejection and long-term improved graft survival of the NIMA-MM sibling kidney transplant. Thus, the maternal DC/EV story is the perfect complement to the phenomenon of NIMA “split tolerance” that manifested itself in both kidney (24) and bone marrow transplantation (28).

Further studies by the Thomson lab added to this new “exosome-based” view of transplantation, this time, in liver transplantation (29). Up until this time, it was generally understood that the liver transplant had tolerogenic properties, thanks to the pioneering work of Calne et al. (30) and Starzl et al. (31). However, the exosome story may underlie the acceptance of hepatic allografts in the absence of immunosuppressive therapy, seen in certain species. In a mouse liver transplant model, where the liver is spontaneously accepted, graft infiltrating (host) DCs rapidly take over the liver from donor DCs (29). Interestingly, a remarkably high proportion (~60%) of graft infiltrating DCs were found to become cross-dressed (XD) with donor MHC 7 days after transplant (29). The source of the MHC I was determined to be hepatocytes. Furthermore, these XD host DCs expressed high levels of PD-L1 and IL-10 and had a strong suppressive effect on the anti-donor MLR. There was no analysis of the relative position on the cell surface of PD-L1 and acquired donor MHC, but the resulting “exhaustion” of graft-infiltrating CD8 T cells suggested that the tolerance effects were predominant.

Identifying the type of donor EV that promotes the cross dressing of recipient cells as well as the cargo contained within the EV are important next steps in characterizing this liver transplant model. In a mouse heart transplant model, Song et al. (32) identified donor-derived exosomes capable of prolonging allograft heart survival. These MHCII, CD9, CD63, and CD81 containing exosomes were able to induce donor antigen-specific Tregs in the recipient (32). Although cross-dressing of lymph node or graft-infiltrating immune cells were not studied, this study does identify some of the key components in the EV modification of the recipient immune system.

Ultimately, the determination of whether the EV cross-dressing model holds true in species where liver transplantation results in rejection rather than tolerance remains to be seen. In humans, ~20% of liver allografts remain tolerant to the recipient following immuno-suppression withdrawal (33), and numerous investigations are underway to identify biomarkers that can predict the successful withdrawal of immunosuppression. By correlating the number of cross-dressed recipient immune cells to transplant rejection episodes, it may be possible to use cross-dressing as a biomarker for prediction of successful immunosuppression withdrawal. Our lab has begun investigating the number of cross-dressed cells in liver transplantation in the peripheral blood in both the deceased and living-donor settings. Although the proportion of cross-dressed cells are rare in the peripheral blood in these patients, we have found that cross-dressed cells have higher surface PD-L1 expression than their non-cross-dressed counterparts. Ongoing studies are investigating the correlation of the frequency of cross-dressed cells with graft survival and rejection episodes.

## The Discovery of IL-35, and Early Hints of its Role in Infectious Tolerance

In 2007, Vignali's lab discovered the novel immuno-suppressive cytokine IL-35 (34), a heterodimer of Ebi3 (35) and the IL12α chain (aka “p35”), both expressed by Treg cells. At first glance, IL-35 appeared to be just another inhibitory cytokine, akin to IL-10 and TGF-β. Its production by Tregs, and its ability to suppress T cell proliferation and various effector functions was undisputed, but it appeared at first to lack any inherent ability to “transfer” regulatory properties to other lymphoid cells. Then came the remarkable discovery by Collison et al. (36) identifying a novel conventional (non-Treg) T cell type that had been induced by IL-35 to become an alternative sort of IL-35-producer. This finding, the first suggesting transfer of suppressive capacity from a Foxp3^+^ Treg to a conventional CD4 T cell (Tconv), revived the “infectious tolerance” idea, 17 years after its original description.

A 2013 review article on the topic of infectious tolerance (37) stated:

“…not only does IL-35 have the ability to directly suppress effector T cell responses, it is also able to expand regulatory responses by propagating infectious tolerance and generating a potent population of IL-35-expressing inducible Tregs.”

That summarized the revitalized status of infectious tolerance in 2013: a small number of Foxp3^+^ Tregs, by producing IL-35, were able to essentially double the (low) level of IL-35 production by converting an equally small number of Tconv cells to become IL-35 producers, dubbed “iTr35 cells” (36). The Vignali lab determined that this “infectious” tolerance step required the formation and use of the heterodimeric IL-35 receptor comprised of both gp130 and IL-12Rβ2 by the “infected” Tconv cell (36). In contrast, homodimers of gp130 or IL-12Rβ2 still allowed for primary suppression, but could not potentiate the conversion of Tconv into iTr35 cells (38) that is required to further potentiate the infectious suppression.

## The Spread of IL-35 Associated Infectious Tolerance

**Figure 2:**
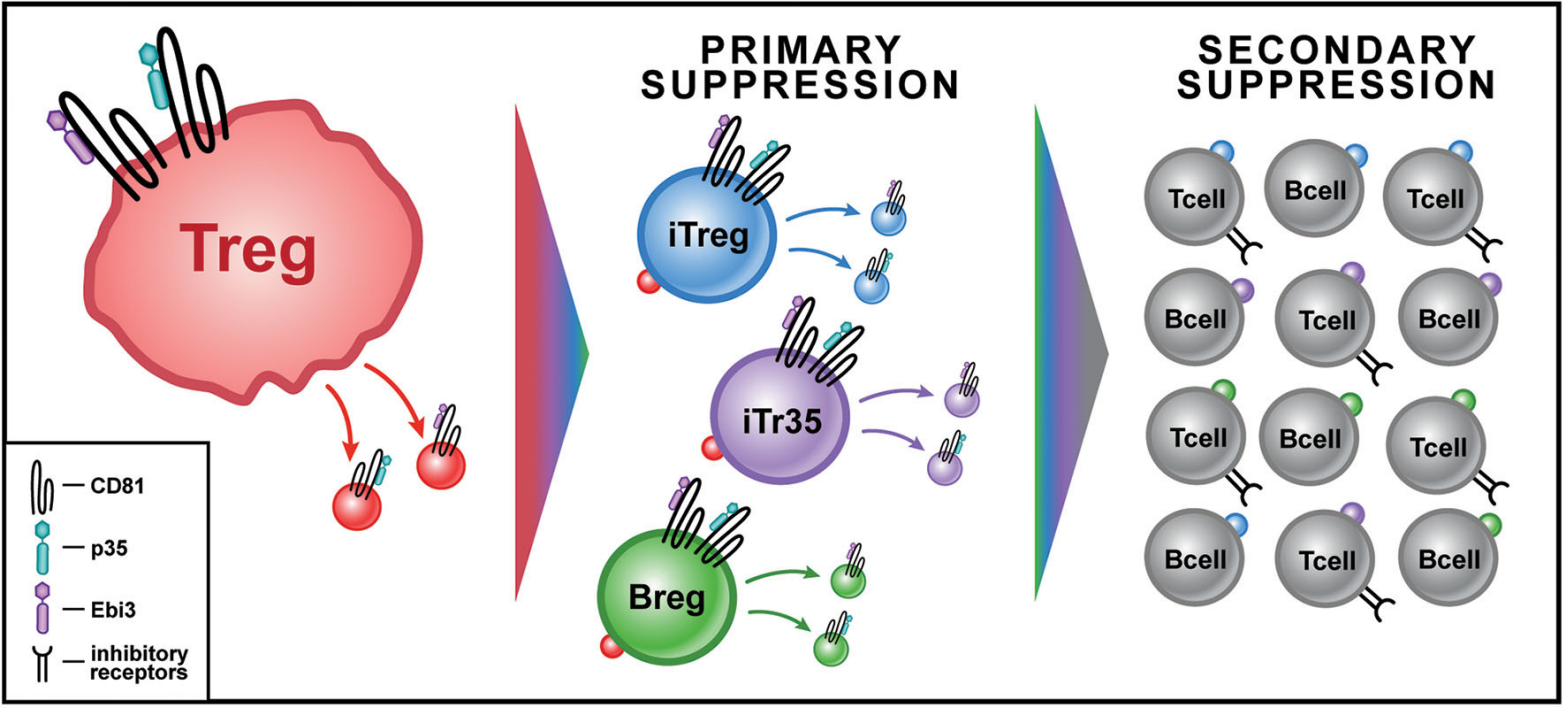
Model for IL-35^+^ EV-Associated Infectious Tolerance from Tregs in Transplantation. Treg cells (red) produce IL-35^+^ EVs in response to antigenic stimulation. IL-35^+^ EVs are bound by lymphoid cells at their IL-35 receptor leading to primary suppression. The primary suppression by IL-35^+^ EVs of conventional CD4 T cells causes some to express Foxp3, becoming inducible Tregs (iTregs-blue) that produce IL35^+^ EVs, while others become non-Foxp3^+^ IL-35^+^ EV producers (iTr35-purple); IL-35^+^ EVs can also induce Bregs (green) to produce their own IL-35^+^ EVs. Secondary suppression by IL35^+^ EV acquiring cells leads to an exponential increase in the cytokine's impact. This can occur through both an increase in inhibitory receptor expression, including PD-1, LAG3, and TIM3, in addition to bystander suppression of local T cell function.

Over the next 6 years (2014–2020), the “conversion” of non-regulatory, conventional lymphocytes into IL-35 producers, began to emerge as a consistent pattern of IL-35-mediated infectious tolerance. The Breg, which up to this point was predominantly associated with production of IL-10 (39, 40), was the first to emerge as a novel producer of IL-35, after induction by Treg-derived IL-35 (41). Furthermore, Treg functions were found to depend in large part upon IL-35 in certain disease contexts. For example, in a mutant mouse strain deficient in low-density lipoprotein receptor (LDL-R), a high cholesterol diet induced a rapid form of atherosclerosis that was dependent on a Th17 response to several autoantigens, including collagen type V (ColV) (42). When the ldlr^−/−^ strain of mice was tolerized with ColV during high cholesterol diet, we found a significant amelioration of the Th17 response to col V and reduction in cardiac lesions. These effects depended not upon IL-10 or TGF-β but overwhelmingly upon IL-35 produced by CD4^+^ Treg, iTr35 cells, and Breg (Figure 2) (43).

The discovery of an association between IL-35 and inhibitory receptor expression in tumor-bearing mouse strains by Turnis et al. (44), extended the range of unique effects of IL-35 to include T cell exhaustion, something that had not been seen previously with IL-10 and TGF-β. How exactly this occurs was left undetermined. The authors noted that: “Curiously, limited, and inconsistent loss of inhibitory receptor (IR) expression was observed in mice treated with anti-IL-35, despite comparable tumor reduction and enhanced T cell activation (data not shown)” (44). They further speculated that “it is possible that antibody-mediated IL-35 neutralization might be incomplete and that the small amount of bioactive IL-35 that remains might induce IR expression” (44). In other words, despite the dramatic effects of IL-35 genetic deletion in Tregs, and anti-tumor effects of anti-IL-35 antibody, the authors could not be certain that the exhaustion of anti-tumor immune T cells was directly the result of IL-35. The process of exhaustion would seem to require something more than simple “single hit” kinetics, i.e., a cytokine binding once to a cytokine receptor. Rather, exhaustion seems to involve multiple, repeated events of T cell signaling. How this could happen if IL-35 were a conventional cytokine, binding only once to its cognate receptor as in the case of IL-10, was not clear.

Studies investigating the function of IL-35 have suggested that it acts differently from other soluble cytokines. For example, recent studies have suggested that IL-35 acts via EVs due to, the ability of ultracentrifugation to separate IL-35 activity from IL-10 secreted by Tregs in culture, the association of IL-35 with the tetraspanin CD81, and the odd proportion of its subunits as produced by Tregs (19). Let's consider each of these factors. First, the ability of all IL-35 activity produced by lymphocytes in culture to be precipitated by 100,000 × g ultracentrifugation, marked this cytokine as clearly different in solubility from IL-10, which remained in the 100,000 × g supernatant. Somehow both subunits of IL-35 wound up in the EV fraction. Second, there was the question of how the 2 different subunits of IL-35, neither of which contains a transmembrane exon, could be tethered either to EV or to the cell surface membrane after EV uptake. This is still a major unsolved mystery. However, one key finding so far is that tetraspanin-association appears to be somewhat specific: ELISAs based on CD81 could detect Treg-derived, IL-35 subunit-containing EVs, while ones based on CD9 [a critical tetraspanin in DC-derived EVs (26)] detection, could not. Experiments are currently underway to determine if CD63, another tetraspanin that has been associated with DC-EVs, could also detect T cell-derived IL-35^+^ EVs, or if the tetraspanin association of IL-35 is truly CD81-specific. Finally, rather than a simple 1:1 subunit ratio predicted to be the active form of IL-35, we found a consistent 2:1 ratio of p35:Ebi3 signal in ELISA assays, and a much stronger band of p35 as compared to Ebi3 on SDS-PAGE after immuno-precipitation of IL-35-CD81 complexes (19). While differing affinities of the Ebi3 and p35 antibodies are the obvious explanation, more work exploring the experimental ratio of Ebi3 and p35 associated with various tetraspanins and its physiological relevance is currently underway.

Because tetraspanins are transmembrane proteins, it is no surprise that the subunits of IL-35, if tetraspanin-bound (45, 46), would tend to migrate to the lymphocyte surface upon uptake of the Treg exosome (19). When re-expressed on the cell surface, CD81-bound subunits were found to generally keep separate from each other. Thus, the EV-decorated lymphoid cell would show predominantly distinct areas of red and green when YFP- and Texas Red fluorescent-tagged antibodies were used to detect each subunit (19). However, the 2 subunits of IL-35 tended to re-associate transiently, based on flashes of yellow seen by Image Stream analysis (19). If real, this transient association could theoretically give rise to transient interactions with the gp130/ IL-12Rβ2 high affinity receptor (IL-35R). If the IL-35R happened to be present on another cell in the vicinity of the EV-altered lymphocyte, it could endow this non-producer lymphocyte, after exosome uptake and re-expression on the cell surface, with the ability to suppress other, “bystander,” T and B cells entering the graft or tumor micro-environment.

In addition, the transient association of IL-35 subunits could have a huge consequence for the non-producer, “decorated” lymphocyte, itself. If tetraspanin-bound subunits of IL-35 from acquired EVs on the surface of a cell could transiently re-associate and bind to the IL-35R, it's therefore possible for a cell to get continuously drenched in EVs containing Ebi3 and/or p35 and have these subunits of IL-35 constantly re-associate and bind the IL-35R on its own cell surface. This is precisely the recipe for T cell exhaustion, multiple, consistent interactions, in this case, IL-35-IL-35R interactions. Indeed, we found that exhaustion, as measured by PD1, LAG3, and TIM3 expression, was found preferentially in those T and B cells that had acquired surface Ebi3 by exosome uptake (19) (Figure 2). It should be noted however that, when tumor rejection was seen in the absence of IL-35, achieved by Ebi3 KO in the tumor host, tumor-infiltrating T cells no longer expressed LAG3 and TIM3, but still expressed PD1, a common activation marker of T helper cells (44). This indicates that LAG3 and TIM3 expression, and more recently, TIGIT (47), are most likely more accurate markers of IL-35-mediated lymphocyte exhaustion than PD1 alone.

The induction by IL-35 of the so-called iTr35 population represented the first reported step in “infectious” tolerance- a doubling of the bio-synthetic capacity of IL-35, as compared to both natural and “induced” Foxp3^+^ Treg populations, alone (36). The later revelation of induction of IL-35-producing CD8 T suppressor cells in prostate cancer patient (48) and in “Bregs” (41) added a second important step. However, these steps still left the lymphocyte population that could produce IL-35 EVs at the level of <0.01% of the total (19). The newly-discovered “passive” conversion of bystander Tconv cells to an immuno-regulatory and exhaustion status, simply by uptake and surface display of EV IL-35 subunits (19), represents ~100-fold magnification of the “infectious tolerance” impact, extending tolerance enforcement to 1% or more of total lymphocytes. Furthermore, at early time points in mouse models of tolerization using donor specific transfusion and anti-CD40L strategies, when IL35^+^ EV-producer cells (iTreg, iTr35, Breg) were barely detectable, Tconv cells passively acquiring surface IL35 expression after EV uptake were easily detected. Such “non-producer,” but surface IL35 subunit-acquiring cells were shown to exert powerful non-specific suppression of other T cells, even as they themselves became gradually exhausted due to chronic self-suppression (19). A model of how this infectious tolerance process might be mediated by EV- associated IL-35 is illustrated in Figure 2.

Recently, a very unusual aspect of IL-35 production by Tregs was suggested by Sawant et al. (49). Rather than the standard dual production of inhibitory cytokines, such as IL-10 and TGFβ, instead they found a time-dependent, “mutually exclusive” pattern of IL-10 and IL-35 production in Treg cells (49). That is to say, both suppressive cytokines could be produced by a single Treg cell, just not at the same time. Furthermore, while both Treg cell-derived IL-10 and IL-35 function cooperatively in driving inhibitory receptor induction on TILs, IL-35 appeared to play a more dominant role in exhaustion. Similarly, Treg cell -restricted deletion of these two cytokines had differential impacts on the tumor micro-environment (TME): IL-10 deletion had a greater impact on limiting effector function and proliferation, whereas, IL-35 deletion impacted exhaustion, and the Treg's ability to limit memory differentiation (49). Given the distinct contributions of each cytokine to tumor escape and graft prolongation, these findings raised a key question: Why is the Treg cell unable to make both IL-35 and IL-10 at the same time? Are the pathways of synthesis somehow fundamentally different?

## IL-35: a Lymphocyte-Derived, EV-associated Cytokine

Since its discovery in 2007 (34, 50), there have been approximately 600 publications on IL-35. All of these assumed that IL-35 was a stable product of Treg, iTr35, and Breg cells, secreted at a 1:1 ratio of subunits p35: Ebi3. There were certain difficulties to this theory, however. For example, Aparicio-Siegmund et al. (51) had no trouble expressing a stable recombinant cytokine IL-12 (p35:p40; IL-12 p70) in bacteria and have it secreted. However, they failed to get IL-35 (p35:Ebi3) similarly produced in the several transfected bacterial cell lines. While both subunits were made, the IL-35 heterodimer was simply not secreted. Even when the two subunits were isolated from bacterial cells and mixed together, they failed to induce signal transduction in Ba/F3 cells expressing IL-12Rβ2 and gp130 (51). The solution to this molecular problem is still not clear. But what is clear is that solution of the structure of native IL-35 will come not as a result of examination of soluble proteins, but by analysis of proteins that associate with EVs (19). Preliminary analysis of a recombinant IL-35 product from Peprotech, which is made in a human kidney cell line at a 1:1 ratio of Ebi3:p35, and sold commercially, indicates that it is composed of both a soluble component (~50%) as well as an EV component (~50%). The latter is precipitate-able at 100,000 × g by ultracentrifugation (WJ Burlingham, unpublished). This suggests that, depending on the ratio of subunit expression, H_2_O soluble forms of human IL-35 may exist. However, there appears to be a preference for exosome association, particularly at the 2:1 ratio of p35: Ebi3 subunits in IL-35 when naturally produced by lymphocytes *in vivo* (19).

## Non-T Cell Sources of IL-35

While a number of reports have implicated Tregs or iTr35 cells as significant IL-35-EV producers, the contributions of other lymphocytes cannot be discounted (Figure 2). B cell subsets (Breg), iNKT, and innate lymphoid cells (ILCs) may also play a significant role in EV related regulation. B cells, including memory B cells, plasma cells, and transitional B cells may all influence the local milieu through EV production. The more recently described regulatory B cell (Breg) which exerts regulatory function through both cytokine production and direct influence, may be one of the most important EV-producing regulatory cells. Unlike the Treg, the Breg exists outside the requirement for antigen processing and “indirect” presentation via MHC molecules, and can respond quickly and deftly to antigen encounter by producing classic regulatory cytokines like IL-10 (39, 40), in addition to EV-associated factors like IL-35 (41).

Is the CD81 tetraspanin absolutely required for the function of IL-35? We don't know the answer at present. What we do know is that the tetraspanin CD9, so important in the DC exosome story of maternal tolerance (26), does not appear to play any role in IL-35 (19). We are currently analyzing both CD81 and CD63 KO mice to see if in either, Tregs retain the ability to produce IL-35, or rather, if the loss of CD81 is not compensated by CD63 or other tetraspanins, leading to an absence of IL-35 in CD81 KO mice. Finally, we would like to leave open the possibility that, in addition to tetraspanin-bound, EV-associated IL-35, there are other “redundant” mechanisms of infectious tolerance, still awaiting discovery. However, the role of IL35 appears to be quite unique in this process. Primary suppression, theoretically, can be the result of a number of non-EV and EV-associated factors. Non-EV associated factors can include IL-10, TGF-β (currently under investigation for EV-association) cell membrane bound activity of the ecto-nucleotidases CD39 and CD73 on myeloid and lymphoid cells to drive hydrolysis of ATP down to adenosine, and other classically secreted cytokines (52–57). EV associated factors include IL-35, and the manipulation of the inflammatory milieu by decreasing extracellular ATP concentrations through EV-associated CD39/CD73 (19, 58). While we were able to show that IL-35 is only one of several possible mechanisms along with CD39/CD73 to facilitate primary immune suppression by EVs, the broad, “infectious” component of secondary immune suppression by EVs was completely abolished by Ebi3 deletion in Tregs (19), delineating rather common primary from the seemingly unique secondary suppression mechanisms related to EVs.

## EVS in Tumor Immunotherapy: Propagating Infectious Tolerance From PD-L1 to IL-35

The ability of EVs, including exosomes, to propagate infectious tolerance has applications not only in transplant immunology and infectious disease, but also in tumor immunology. In addition to the various mechanisms described above in which regulatory immune populations can propagate infectious tolerance, the generation of tumor-derived exosomes across a variety of malignancies can be used not only as biomarkers of disease progression and metastasis, but also serve to both directly mediate immune suppression of effector populations as well as propagate infectious tolerance (see below).

This presents both a challenge, as well as an opportunity, for tumor immunotherapy. While both tumor-derived and host-derived exosomes can mediate immune suppression, perhaps they also can be harnessed to direct and enhance the anti-tumor immune response. For example, the phenomenon of acute rejection of the MHC- and minor H-mismatched allograft, long a fixture of transplant immunology, has recently been re-evaluated. It was shown not to be the result of the so-called “direct” pathway of alloreactivity, a powerful activity measured *in vitro* by the MLR, or “mixed lymphocyte reaction.” Instead, *in vivo* it turned out to be the result of “semi-direct” allo-reactivity: whereby a tiny number of graft dendritic cells caused a tremendous amplification of the number of host allo-reactive T cells engaged in the immune response, by exosomes they released that were taken up widely by host dendritic cells (20, 21). In regards to cancer immunotherapy, perhaps EVs of the right variety could actually amplify, rather than suppress anti-tumor immunity.

One of the most well-characterized mechanisms by which tumor-derived exosomes induce tolerance within the tumor microenvironment is by taking up various suppressive ligands that can directly bind to target cells, and then transporting these molecules both locally as well as systemically where they can suppress effector immune responses. One of the most well-characterized examples of this direct suppression is tumor-derived PD-L1^+^ exosomes, which can suppress cytotoxic CD8^+^ T cell responses to promote tumor growth (59). Not only can these exosomes directly suppress effector responses, but they can also serve as a means or propagating infectious tolerance, by transferring PD-L1 to other tumor and immune populations (60). This also has directly relevance to patient care, as patients with decreased frequency of PD-L1^+^ exosomes prior to treatment predict for clinical responsiveness to PD-1 blockade (59). Furthermore, blockade of exosomal PD-L1 can not only suppress tumor growth, and in fact works additively with PD-L1 blockade (61). This suggests that these exosomes containing and coated with PD-L1 may be suppressing anti-tumor immunity using a separate mechanism than that targeted by systemic PD-L1 blockade. Similar results have also been observed for other molecules coating the surface tumor-derived exosomes, such as Hsp72 [which induces STAT3 signaling to promote the expansion and suppressive function of MDSCs (62)], as well as surface-bound Gal9 (63) and Fas ligand (64) exosomes that can directly suppress effector T cell activity.

Another means by which tumor-cell derived exosomes can promote tolerogenic responses in the TME is by targeting the metabolism and activity of effector populations through the generation and accumulation of adenosine (65–67). In a variety of malignancies, tumor-derived EVs, can be coated with the ecto-nucleotidases CD39 and CD73, which can suppress anti-tumor effector responses as well as promote the enhanced activity of regulatory T cells through extracellular ATP manipulation (68–70). As this is a mechanism by which CD4^+^ Tregs mediate suppression and the propagation of primary infectious tolerance (71), it is also likely that this is a means by which tumor-derived EVs can mediate infectious tolerance within the TME.

In addition to their ability to directly target the activity of effector populations within the tumor microenvironment, tumor-derived exosomes can mediate the suppression of anti-tumor immunity by propagating infectious tolerance via immunosuppressive cytokines. Tumor-derived exosomes were found to convert conventional CD4^+^CD25^neg^ T cells into a potent suppressive population of CD4^+^CD25^hi^ Foxp3^+^ Tregs, with increased expression of TGF-β, IL-10, CTLA-4, and enhanced suppressive activity against effector responses, which can be reversed through the inhibition of TGF-β or IL-10 (72, 73). Similar results have been observed in several other malignancies, including in malignant effusions where TGF-β propagates infectious tolerance (74), in leukemia blasts where TGF-β suppress NK cell effector activity (75), and in breast cancer where tumor-derived PGE2 and TGF-β exosomes convert myeloid cells to MDSCs to promote tumor progression and infectious tolerance (76). These data illustrate how immunosuppressive cytokines play a central role in the propagation of infectious tolerance in the tumor microenvironment.

With a clearly established role for tumor-derived exosomes utilizing immunosuppressive cytokines to mediate tolerance, a critical outstanding question is the role of IL-35 in immune suppression mediated by exosomes in the tumor microenvironment. Given our previous observations that human tumor antigen-specific regulatory populations can utilize IL-35, and that they are distinct from those utilizing TGF-β or IL-10 (48), then if it can be shown that such human Tregs also produce EV associated IL-35, similar to mice (19), it suggests that these IL-35^+^ Tregs may be able to propagate infectious tolerance via a previously uninvestigated mechanism such as IL-35^+^ EVs. While IL-35–producer cells were found at extremely low frequency within the blood of cancer patients, the fact that they could still suppress the detection of anti-tumor effector immune responses prior to immunotherapeutic intervention might speak to the power of the exosome amplification mechanism described for murine IL-35 (19). Additionally, just as PDL1 blockade alone does not target the activity of PD-L1^+^ exosomes, antibody blockade of Ebi3/IL-35 was not sufficient to reverse immunosuppression in murine cancer models (44), which may suggest the possibility that a strategy to target Ebi3^+^ EVs could more effectively reverse suppression within the tumor microenvironment.

## Conclusions

As detailed above, research on the role of EVs in promoting transplantation tolerance, and limiting anti-tumor immunity, is still in an early stage. Immunological tolerance is multi-faceted, and the role of EVs in infectious tolerance is one possible mechanism to describe the establishment and maintenance of this process. The parallel observations of PDL1-, CTLA-4-, and CD39/CD73-based tolerance in both fields raise the possibility that overcoming immune suppression and releasing tumor cytotoxicity in cancer, as well as enforcing immune regulation in transplant recipients, is becoming increasingly about controlling the EV content of the tumor and transplant micro-environment. The challenge raised by the discovery that IL-35 is in fact not a H_2_0-soluble, stable suppressive cytokine but rather is carried on EVs by tetraspanin(s), delivering “multi-hit” rather than single-hit suppressive signals by repetitive binding to its receptor on T and B cells. This raises new questions for immunologists in both fields. The tolerance possibilities in well-(HLA) matched transplants, and eventually in poorly-matched transplants, can be enhanced by greater understanding of pathways of EV-mediated suppression, particularly those pathways leading to exhaustion of host T and B cells. On the other hand, tumor immunologists, who have already begun to explore anti-inflammatory aspects of exosomes that limit immunotherapy, can now add IL-35 to their “EV” target list. By adding IL-35 along with PD1/PDL1 to the “EV” category of tumor immune escape mechanisms, tumor immuno-therapists may now focus upon developing even more effective new weapons against tumors. These new weapons will be based on disarming inhibitory EV production, preventing uptake by host T and dendritic cells, and releasing anti-tumor CD8 killer T cells from immune suppression and exhaustion. T cell exhaustion may in fact be a unique aspect of the IL-35 cytokine (44) due to its EV nature (19), that may be reversible by future exosome targeting strategies. Overall, there seems to be a promising future for EV-based therapies as we look forward to making transplant tolerance more common as a clinical strategy, and using EV-targeting strategies to improve upon the substantial gains made by tumor immunotherapy to make tumor rejection by the host a more common therapeutic result.

## Author Contributions

WB designed the review. DA wrote the section on liver transplant. BO and DM contributed the section on exosomes and tumor immunity. JS wrote the key reference and provided historical perspective. All authors contributed to the article and approved the submitted version.

## Conflict of Interest

DM is scientific founder, consultant at, has received ownership interest (including patents) in, and is a consultant/advisory board for Madison Vaccines, Inc. BO has intellectual property licensed through Madison Vaccines, Inc. The remaining authors declare that the research was conducted in the absence of any commercial or financial relationships that could be construed as a potential conflict of interest.

## References

[1] Gershon RK Kondo K. Infectious immunological tolerance. Immunology. (1971) 21:903–14. 4943147 PMC1408252

[2] Qin S Cobbold SP Pope H Elliott J Kioussis D Davies J. 'Infectious' transplantation tolerance. Science. (1993) 259:974–7. 10.1126/science.8094901 8094901

[3] Brunkow ME Jeffery EW Hjerrild KA Paeper B Clark LB Yasayko SA. Disruption of a new forkhead/winged-helix protein, scurfin, results in the fatal lymphoproliferative disorder of the scurfy mouse. Nat Genet. (2001) 27:68–73. 10.1038/83784 11138001

[4] Sakaguchi S Sakaguchi N Asano M Itoh M Toda M. Immunologic self-tolerance maintained by activated T cells expressing IL-2 receptor alpha-chains (CD25). Breakdown of a single mechanism of self-tolerance causes various autoimmune diseases. J Immunol. (1995) 155:1151–64. 7636184

[5] Chatila TA Blaeser F Ho N Lederman HM Voulgaropoulos C Helms C. JM2, encoding a fork head-related protein, is mutated in X-linked autoimmunity-allergic disregulation syndrome. J Clin Invest. (2000) 106:R75–81. 10.1172/JCI11679 11120765 PMC387260

[6] Waldmann H Adams E Fairchild P Cobbold S. Infectious tolerance and the long-term acceptance of transplanted tissue. Immunol Rev. (2006) 212:301–13. 10.1111/j.0105-2896.2006.00406.x 16903922

[7] Cobbold SP Adams E Graca L Daley S Yates S Paterson A. Immune privilege induced by regulatory T cells in transplantation tolerance. Immunol Rev. (2006) 213:239–55. 10.1111/j.1600-065X.2006.00428.x 16972908

[8] Sakaguchi S Sakaguchi N Asano M Itoh M Toda M. Immunologic self-tolerance maintained by activated T cells expressing IL-2 receptor alpha-chains (CD25). Breakdown of a single mechanism of self-tolerance causes various autoimmune diseases. J Immunol. (1995) 155:1151–64. 7636184

[9] Fontenot JD Gavin MA Rudensky AY. Foxp3 programs the development and function of CD4+CD25+ regulatory T cells. Nat Immunol. (2003) 4:330–6. 10.1038/ni904 12612578

[10] Hall BM Pearce NW Gurley KE Dorsch SE. Specific unresponsiveness in rats with prolonged cardiac allograft survival after treatment with cyclosporine. III. Further characterization of the CD4+ suppressor cell and its mechanisms of action. J Exp Med. (1990) 171:141–57. 10.1084/jem.171.1.141 2136906 PMC2187663

[11] Ilano AL Spinelli A Gurley KE Strober S Hall BM. Induction of unresponsiveness to organ allografts. A comparison of different immunosuppressive protocols in DA and WF strains of rats. Transplantation. (1991) 51:905–9. 10.1097/00007890-199104000-00034 1901677

[12] de Larco JE Todaro GJ. Growth factors from murine sarcoma virus-transformed cells. Proc Natl Acad Sci USA. (1978) 75:4001–5. 10.1073/pnas.75.8.4001 211512 PMC392918

[13] Todaro GJ De Larco JE. Growth factors produced by sarcoma virus-transformed cells. Cancer Res. (1978) 38(11 Pt. 2):4147–54. 212188

[14] Roberts AB Anzano MA Lamb LC Smith JM Sporn MB. New class of transforming growth factors potentiated by epidermal growth factor: isolation from non-neoplastic tissues. Proc Natl Acad Sci USA. (1981) 78:5339–43. 10.1073/pnas.78.9.5339 6975480 PMC348740

[15] Moses HL Roberts AB Derynck R. The discovery and early days of TGF-β: a historical perspective. Cold Spring Harb Perspect Biol. (2016) 8:a021865. 10.1101/cshperspect.a021865 27328871 PMC4930926

[16] Fiorentino DF Bond MW Mosmann TR. Two types of mouse T helper cell. IV. Th2 clones secrete a factor that inhibits cytokine production by Th1 clones. J Exp Med. (1989) 170:2081–95. 10.1084/jem.170.6.2081 2531194 PMC2189521

[17] Saraiva M O'Garra A. The regulation of IL-10 production by immune cells. Nat Rev Immunol. (2010) 10:170–81. 10.1038/nri2711 20154735

[18] Gravano DM Vignali DA. The battle against immunopathology: infectious tolerance mediated by regulatory T cells. Cell Mol Life Sci. (2012) 69:1997–2008. 10.1007/s00018-011-0907-z 22205213 PMC3353028

[19] Sullivan JA Tomita Y Jankowska-Gan E Lema DA Arvedson MP Nair A. Treg-cell-derived IL-35-coated extracellular vesicles promote infectious tolerance. Cell Rep. (2020) 30:1039–51.e5. 10.1016/j.celrep.2019.12.081 31995748 PMC7042971

[20] Marino J Babiker-Mohamed M Crosby-Bertorini P Paster JT LeGuern C Germana S. Donor exosomes rather than passenger leukocytes initiate alloreactive T cell responses after transplantation. Sci Immunol. (2016) 1:aaf8759. 10.1126/sciimmunol.aaf8759 27942611 PMC5142759

[21] Liu Q Rojas-Canales DM Divito SJ Shufesky WJ Stolz DB Erdos G. Donor dendritic cell-derived exosomes promote allograft-targeting immune response. J Clin Invest. (2016) 126:2805–20. 10.1172/JCI84577 27348586 PMC4966303

[22] Larsen CP Morris PJ Austyn JM. Migration of dendritic leukocytes from cardiac allografts into host spleens. J ExpMed. (1990) 171:307–14. 10.1084/jem.171.1.307 2404081 PMC2187651

[23] Claas FH Gijbels Y van Der Velden-de Munck J van Rood JJ. Induction of B cell unresponsiveness to noninherited maternal HLA antigens during fetal life. Science. (1988) 241:1815–7. 10.1126/science.3051377 3051377

[24] Burlingham WJ Grailer AP Heisey DM Claas FH Norman D Mohanakumar T. The effect of tolerance to noninherited maternal HLA antigens on the survival of renal transplants from sibling donors [see comments]. N Engl J Med. (1998) 339:1657–64. 10.1056/NEJM199812033392302 9834302

[25] van Rood JJ Loberiza FRJ Zhang MJ Oudshoorn M Claas F Cairo MS Effect of tolerance to noninherited maternal antigens on the occurrence of graft-versus-host disease after bone marrow transplantation from a parent or an HLA-haploidentical sibling. Blood. (2002) 99:1572–7. 10.1182/blood.V99.5.1572 11861270

[26] Bracamonte-Baran W Florentin J Zhou Y Jankowska-Gan E Haynes WJ Zhong W. Modification of host dendritic cells by microchimerism-derived extracellular vesicles generates split tolerance. Proc Natl Acad Sci USA. (2017) 114:1099–104. 10.1073/pnas.1618364114 28096390 PMC5293109

[27] Morelli AE Bracamonte-Baran W Burlingham WJ. Donor-derived exosomes: the trick behind the semidirect pathway of allorecognition. Curr Opin Organ Transplant. (2017) 22:46–54. 10.1097/MOT.0000000000000372 27898464 PMC5407007

[28] van Rood JJ Stevens CE Smits J Carrier C Carpenter C Scaradavou A. Reexposure of cord blood to noninherited maternal HLA antigens improves transplant outcome in hematological malignancies. Proc Natl Acad Sci USA. (2009) 106:19952–7. 10.1073/pnas.0910310106 19901324 PMC2775036

[29] Ono Y Perez-Gutierrez A Nakao T Dai H Camirand G Yoshida O. Graft-infiltrating PD-L1hi cross-dressed dendritic cells regulate anti-donor T cell responses in mouse liver transplant tolerance. Hepatology. (2017) 67:1499–515. 10.1097/01.tp.0000520343.32573.7c 28921638 PMC5856603

[30] Calne RY Sells RA Pena JR Davis DR Millard PR Herbertson BM. Induction of immunological tolerance by porcine liver allografts. Nature. (1969) 223:472–6. 10.1038/223472a0 4894426

[31] Starzl TE Demetris AJ Trucco M Ramos H Zeevi A Rudert WA. Systemic chimerism in human female recipients of male livers. Lancet. (1992) 340:876–7. 10.1016/0140-6736(92)93286-V 1357298 PMC3184834

[32] Song J Huang J Chen X Teng X Song Z Xing Y Donor-derived exosomes induce specific regulatory T cells to suppress immune inflammation in the allograft heart. Sci Rep. (2016) 7:20077 10.1038/srep46389 26822278 PMC4731812

[33] Londono MC Rimola A O'Grady J Sanchez-Fueyo A. Immunosuppression minimization vs complete drug withdrawal in liver transplantation. J Hepatol. (2013) 59:872–9. 10.1016/j.jhep.2013.04.003 23578883

[34] Collison LW Workman CJ Kuo TT Boyd K Wang Y Vignali KM. The inhibitory cytokine IL-35 contributes to regulatory T-cell function. Nature. (2007) 450:566–9. 10.1038/nature06306 18033300

[35] Devergne O Hummel M Koeppen H Le Beau MM Nathanson EC Kieff E. A novel interleukin-12 p40-related protein induced by latent Epstein-Barr virus infection in B lymphocytes. J Virol. (1996) 70:1143–53. 10.1128/JVI.70.2.1143-1153.1996 8551575 PMC189923

[36] Collison LW Chaturvedi V Henderson AL Giacomin PR Guy C Bankoti J. IL-35-mediated induction of a potent regulatory T cell population. Nat Immunol. (2010) 11:1093–101. 10.1038/ni.1952 20953201 PMC3008395

[37] Olson BM Sullivan JA Burlingham WJ. Interleukin 35: a key mediator of suppression and the propagation of infectious tolerance. Front Immunol. (2013) 4:315. 10.3389/fimmu.2013.00315 24151492 PMC3798782

[38] Collison LW Delgoffe GM Guy CS Vignali KM Chaturvedi V Fairweather D. The composition and signaling of the IL-35 receptor are unconventional. Nat Immunol. (2012) 13:290–9. 10.1038/ni.2227 22306691 PMC3529151

[39] Yanaba K Bouaziz JD Haas KM Poe JC Fujimoto M Tedder TF. A regulatory B cell subset with a unique CD1dhiCD5+ phenotype controls T cell-dependent inflammatory responses. Immunity. (2008) 28:639–50. 10.1016/j.immuni.2008.03.017 18482568

[40] Piper CJM Rosser EC Oleinika K Nistala K Krausgruber T Rendeiro AF. Aryl hydrocarbon receptor contributes to the transcriptional program of IL-10-producing regulatory B cells. Cell Rep. (2019) 29:1878–92.e7. 10.1016/j.celrep.2019.10.018 31722204 PMC6856759

[41] Wang RX Yu CR Dambuza IM Mahdi RM Dolinska MB Sergeev YV. Interleukin-35 induces regulatory B cells that suppress autoimmune disease. Nat Med. (2014) 20:633–41. 10.1038/nm.3554 24743305 PMC4048323

[42] Dart ML Jankowska-Gan E Huang G Roenneburg DA Keller MR Torrealba JR. Interleukin-17-dependent autoimmunity to collagen type V in atherosclerosis. Circ Res. (2010) 107:1106–16. 10.1161/CIRCRESAHA.110.221069 20814021 PMC3010213

[43] Park AC Huang G Jankowska-Gan E Massoudi D Kernien JF Vignali DA. Mucosal administration of collagen V ameliorates atherosclerotic plaque burden by inducing IL-35-dependent tolerance. J Biol Chem. (2015) 291:3359–70. 10.1074/jbc.M115.681882 26721885 PMC4751380

[44] Turnis ME Sawant DV Szymczak-Workman AL Andrews LP Delgoffe GM Yano H. Interleukin-35 limits anti-tumor immunity. Immunity. (2016) 44:316–29. 10.1016/j.immuni.2016.01.013 26872697 PMC4758699

[45] Shoham T Rajapaksa R Kuo CC Haimovich J Levy S. Building of the tetraspanin web: distinct structural domains of CD81 function in different cellular compartments. Mol Cell Biol. (2006) 26:1373–85. 10.1128/MCB.26.4.1373-1385.2006 16449649 PMC1367195

[46] Levy S. Function of the tetraspanin molecule CD81 in B and T cells. Immunol Res. (2014) 58:179–85. 10.1007/s12026-014-8490-7 24522698

[47] Riquelme P Haarer J Kammler A Walter L Tomiuk S Ahrens N. TIGIT(+) iTregs elicited by human regulatory macrophages control T cell immunity. Nat Commun. (2018) 9:2858. 10.1038/s41467-018-05167-8 30030423 PMC6054648

[48] Olson BM Jankowska-Gan E Becker JT Vignali DA Burlingham WJ McNeel DG Human prostate tumor antigen-specific CD8+ regulatory T cells are inhibited by CTLA-4 or IL-35 blockade. J Immunol. (2012) 189:5590–601. 10.4049/jimmunol.1201744 23152566 PMC3735346

[49] Sawant DV Yano H Chikina M Zhang Q Liao M Liu C. Adaptive plasticity of IL-10(+) and IL-35(+) Treg cells cooperatively promotes tumor T cell exhaustion. Nat Immunol. (2019) 20:724–35. 10.1038/s41590-019-0346-9 30936494 PMC6531353

[50] Niedbala W Wei XQ Cai B Hueber AJ Leung BP McInnes IB. IL-35 is a novel cytokine with therapeutic effects against collagen-induced arthritis through the expansion of regulatory T cells and suppression of Th17 cells. Eur J Immunol. (2007) 37:3021–9. 10.1002/eji.200737810 17874423

[51] Aparicio-Siegmund S Moll JM Lokau J Grusdat M Schroder J Plohn S. Recombinant p35 from bacteria can form interleukin (IL-)12, but not IL-35. PLoS ONE. (2014) 9:e107990. 10.1371/journal.pone.0107990 25259790 PMC4178060

[52] Sullivan JA Jankowska-Gan E Hegde S Pestrak MA Agashe VV Park AC. Th17 responses to collagen type V, kα1-Tubulin, and vimentin are present early in human development and persist throughout life. Am J Transplant. (2017) 17:944–56. 10.1111/ajt.14097 27801552 PMC5626015

[53] Sullivan JA Jankowska-Gan E Shi L Roenneburg D Hegde S Greenspan DS. Differential requirement for P2X7R function in IL-17 dependent vs IL-17 independent cellular immune responses. Am J Transplant. (2014) 14:1512–22. 10.1111/ajt.12741 24866539 PMC4295495

[54] Longhi MS Vuerich M Kalbasi A Kenison JE Yeste A Csizmadia E. Bilirubin suppresses Th17 immunity in colitis by upregulating CD39. JCI Insight. (2017) 2:e92791. 10.1172/jci.insight.92791 28469075 PMC5414551

[55] Samudra AN Dwyer KM Selan C Freddi S Murray-Segal L Nikpour M. CD39 and CD73 activity are protective in a mouse model of antiphospholipid antibody-induced miscarriages. J Autoimmun. (2018) 88:131–8. 10.1016/j.jaut.2017.10.009 29103803

[56] Warren CA Calabrese GM Li Y Pawlowski SW Figler RA Rieger J. Effects of adenosine A(2)A receptor activation and alanyl-glutamine in *Clostridium* difficile toxin-induced ileitis in rabbits and cecitis in mice. BMC Infect Dis. (2012) 12:13. 10.1186/1471-2334-12-13 22264229 PMC3323464

[57] Eltzschig HK Sitkovsky MV Robson SC. Purinergic signaling during inflammation. N Engl J Med. (2012) 367:2322–33. 10.1056/NEJMra1205750 23234515 PMC3675791

[58] Allard B Longhi MS Robson SC Stagg J. The ectonucleotidases CD39 and CD73: novel checkpoint inhibitor targets. Immunol Rev. (2017) 276:121–44. 10.1111/imr.12528 28258700 PMC5338647

[59] Chen G Huang AC Zhang W Zhang G Wu M Xu W. Exosomal PD-L1 contributes to immunosuppression and is associated with anti-PD-1 response. Nature. (2018) 560:382–6. 10.1038/s41586-018-0392-8 30089911 PMC6095740

[60] Yang Y Li CW Chan LC Wei Y Hsu JM Xia W. Exosomal PD-L1 harbors active defense function to suppress T cell killing of breast cancer cells and promote tumor growth. Cell Res. (2018) 28:862–4. 10.1038/s41422-018-0060-4 29959401 PMC6082826

[61] Poggio M Hu T Pai CC Chu B Belair CD Chang A. Suppression of exosomal PD-L1 induces systemic anti-tumor immunity and memory. Cell. (2019) 177:414–27.e13. 10.1016/j.cell.2019.02.016 30951669 PMC6499401

[62] Chalmin F Ladoire S Mignot G Vincent J Bruchard M Remy-Martin JP. Membrane-associated Hsp72 from tumor-derived exosomes mediates STAT3-dependent immunosuppressive function of mouse and human myeloid-derived suppressor cells. J Clin Invest. (2010) 120:457–71. 10.1172/JCI40483 20093776 PMC2810085

[63] Klibi J Niki T Riedel A Pioche-Durieu C Souquere S Rubinstein E. Blood diffusion and Th1-suppressive effects of galectin-9-containing exosomes released by Epstein-Barr virus-infected nasopharyngeal carcinoma cells. Blood. (2009) 113:1957–66. 10.1182/blood-2008-02-142596 19005181

[64] Taylor DD Gercel-Taylor C Lyons KS Stanson J Whiteside TL. T-cell apoptosis and suppression of T-cell receptor/CD3-zeta by fas ligand-containing membrane vesicles shed from ovarian tumors. Clin Cancer Res. (2003) 9:5113–9. 10.4049/d.2003 14613988

[65] Ohta A Sitkovsky M. Extracellular adenosine-mediated modulation of regulatory T cells. Front Immunol. (2014) 5:304. 10.3389/fimmu.2014.00304 25071765 PMC4091046

[66] Ernst PB Garrison JC Thompson LF. Much ado about adenosine: adenosine synthesis and function in regulatory T cell biology. J Immunol. (2010) 185:1993–8. 10.4049/jimmunol.1000108 20686167 PMC3036969

[67] Linden J Cekic C. Regulation of lymphocyte function by adenosine. Arterioscler Thromb Vasc Biol. (2012) 32:2097–103. 10.1161/ATVBAHA.111.226837 22772752 PMC4476649

[68] Clayton A Al-Taei S Webber J Mason MD Tabi Z. Cancer exosomes express CD39 and CD73, which suppress T cells through adenosine production. J Immunol. (2011) 187:676–83. 10.4049/jimmunol.1003884 21677139

[69] Muller L Mitsuhashi M Simms P Gooding WE Whiteside TL. Tumor-derived exosomes regulate expression of immune function-related genes in human T cell subsets. Sci Rep. (2016) 6:20254. 10.1038/srep20254 26842680 PMC4740743

[70] Schuler PJ Saze Z Hong CS Muller L Gillespie DG Cheng D Human CD4+ CD39+ regulatory T cells produce adenosine upon co-expression of surface CD73 or contact with CD73+ exosomes or CD73+ cells. Clin Exp Immunol. (2014) 177:531–43. 10.1111/cei.12354 24749746 PMC4226604

[71] Smyth LA Ratnasothy K Tsang JY Boardman D Warley A Lechler R. CD73 expression on extracellular vesicles derived from CD4+ CD25+ Foxp3+ T cells contributes to their regulatory function. Eur J Immunol. (2013) 43:2430–40. 10.1002/eji.201242909 23749427

[72] Muller L Simms P Hong CS Nishimura MI Jackson EK Watkins SC. Human tumor-derived exosomes (TEX) regulate Treg functions via cell surface signaling rather than uptake mechanisms. Oncoimmunology. (2017) 6:e1261243. 10.1080/2162402X.2016.1261243 28919985 PMC5593709

[73] Szajnik M Czystowska M Szczepanski MJ Mandapathil M Whiteside TL. Tumor-derived microvesicles induce, expand and up-regulate biological activities of human regulatory T cells (Treg). PLoS ONE. (2010) 5:e11469. 10.1371/journal.pone.0011469 20661468 PMC2908536

[74] Wada J Onishi H Suzuki H Yamasaki A Nagai S Morisaki T. Surface-bound TGF-beta1 on effusion-derived exosomes participates in maintenance of number and suppressive function of regulatory T-cells in malignant effusions. Anticancer Res. (2010) 30:3747–57. Available online at: http://ar.iiarjournals.org/content/30/9/3747.full 20944164

[75] Szczepanski MJ Szajnik M Welsh A Whiteside TL Boyiadzis M. Blast-derived microvesicles in sera from patients with acute myeloid leukemia suppress natural killer cell function via membrane-associated transforming growth factor-beta1. Haematologica. (2011) 96:1302–9. 10.3324/haematol.2010.039743 21606166 PMC3166100

[76] Xiang X Poliakov A Liu C Liu Y Deng ZB Wang J. Induction of myeloid-derived suppressor cells by tumor exosomes. Int J Cancer. (2009) 124:2621–33. 10.1002/ijc.24249 19235923 PMC2757307

